# Teacher motivational practices and their perceived influence on L2 motivation in Libyan secondary EFL classrooms

**DOI:** 10.3389/fpsyg.2026.1701372

**Published:** 2026-02-18

**Authors:** Emre Debreli, Hana Abdulqadir Almabrouk Abdulqadir

**Affiliations:** Faculty of Education, Cyprus International University, Nicosia, Cyprus

**Keywords:** ELT, instructional strategies, L2 confidence, L2 engagement, learner motivation, Libya, secondary education, teaching practices

## Abstract

Learner motivation is a critical factor in the success of foreign language learning. This study investigated the instructional practices employed by Libyan secondary school EFL teachers and their perceived influence on learners’ motivation, engagement, and confidence. In this study, learner motivation, engagement, and confidence are operationalised exclusively through teachers’ perceptions rather than direct learner self-reports. A quantitative survey design was adopted, drawing on responses from 250 teachers across five secondary schools. A researcher-developed Likert-scale questionnaire, validated through expert review and pilot testing, measured ten dimensions of instructional practice. Descriptive and inferential analyses, including t-tests, ANOVA, correlation, and regression, were conducted using SPSS. Results indicated that teacher feedback, classroom interaction, and autonomy support were the strongest predictors of teacher-reported learner motivation, while cultural responsiveness and classroom management were weaker and less reliable indicators. Younger teachers reported greater use of innovative, student-centered approaches than their more experienced counterparts. These findings align with Self-Determination Theory and Teacher Expectancy Theory, underscoring the importance of competence, relatedness, and autonomy in fostering motivation. The study contributes empirical evidence from a rarely studied context, offering implications for teacher professional development, curriculum policy, and future research on motivational practices in EFL classrooms.

## 1 Introduction

English has become a central language for global communication, enabling cultural exchange and providing opportunities for academic and professional advancement (Ly, 2024; Rao, 2019). As Crystal (2003) and Graddol (2006) have noted, the global spread of English has positioned the language not only as a tool for communication but also as a marker of socioeconomic mobility and identity construction. In contexts where English is taught as a foreign language (EFL), the effectiveness of teaching practices is particularly significant. Libya is one such context, where the education system faces persistent challenges due to socio-political transitions and limited resources. Within this environment, learners’ motivation to learn English as a second or foreign language (often termed L2 motivation) at the secondary level is critical, as it shapes both their immediate success in language learning and their long-term attitudes toward English.

Recent pedagogical approaches have shifted away from teacher-centered instruction, emphasizing instead student-centered practices that cultivate autonomy, critical thinking, and active engagement (Hamre and Pianta, 2001; Wang et al., 2019). Instructors are now seen as facilitators who create learning environments that stimulate learners’ interest and responsibility. Motivation, therefore, is not only an individual learner attribute but also closely tied to how teachers structure classroom experiences and interact with their students (Lamb, 2017; Sase et al., 2015). However, in the domain of second language acquisition (SLA), motivation is conceptualized as a distinct construct—referred to as L2 motivation—that encompasses learners’ desires, goals, and efforts specifically directed toward language learning (Dörnyei, 2014; Ushioda, 2011). This construct differs from general academic motivation because of its affective, cognitive, and sociocultural dimensions, including identity, imagined communities, and perceptions of linguistic competence (Gardner, 2010; Lamb, 2017).

Research consistently demonstrates that teacher behaviors—such as encouragement, feedback, and the use of varied instructional strategies—play a decisive role in sustaining L2 learners’ motivation and persistence (Begum et al., 2023; Ly, 2024). Studies in SLA also show that L2 engagement and L2 confidence are influenced by teachers’ instructional practices, particularly when those practices support learners’ sense of autonomy, competence, and relatedness (MacIntyre et al., 1998; Mercer and Dörnyei, 2020). Although learner motivation has been widely studied, the Libyan secondary school context remains underexplored. Previous research has primarily highlighted structural difficulties such as outdated teaching methods and insufficient resources (Pathan and Marayi, 2016), yet less attention has been given to how teachers’ day-to-day practices affect learners’ L2 motivation, L2 engagement, and confidence. Moreover, the majority of existing studies rely heavily on sources published more than a decade ago, which limits their relevance to current debates. Recent developments in L2 motivation research, including the L2 Motivational Self System and perspectives on multilingual learner identities (Dörnyei, 2009; Ueki and Takeuchi, 2013), have not yet been systematically applied to the Libyan EFL setting.

Contemporary perspectives on language learning motivation—including work grounded in Self-Determination Theory (Deci and Ryan, 1985; Ryan and Deci, 2020) and Teacher Expectancy Theory (Rosenthal and Jacobson, 1968)—offer important insights into how teacher behaviors shape motivational outcomes in L2 classrooms. Yet, these theoretical frameworks remain underutilized in empirical research in Libya, despite their relevance for understanding how teachers can enhance learners’ confidence, engagement, and willingness to invest effort in L2 learning.

This study aims to address this gap by examining the relationship between teacher behaviors, instructional practices, and L2 learner motivation, engagement, and confidence in Libyan secondary schools. The findings are expected to inform teacher development initiatives and contribute to a more current and contextually relevant understanding of motivational dynamics in EFL classrooms. Accordingly, the present study adopts a teacher-report perspective, focusing on how EFL teachers perceive the influence of their instructional practices on learners’ motivation, engagement, and confidence, rather than measuring learners directly. Specifically, the research is guided by the following questions:

*RQ1*: How do Libyan secondary school EFL teachers perceive the relationship between their instructional practices and learners’ L2 motivation and engagement?

*RQ2*: Which instructional practices do EFL teachers perceive as most strongly associated with learners’ L2 confidence in Libyan secondary schools?

## 2 Literature review

### 2.1 Teachers’ conduct and student motivation

A substantial body of research underscores the decisive role of teachers’ conduct in shaping students’ motivation and subsequent academic achievement. Teachers’ behaviors—ranging from their instructional strategies to their interpersonal interactions—serve as powerful signals that shape how students perceive themselves and their learning environment. When teachers demonstrate supportive, encouraging, and engaging behaviors, they cultivate classroom climates that foster self-confidence, resilience, and a willingness to persist in the face of challenges (Abid and Akhtar, 2020; Reeve, 2006). Motivated learners not only tend to achieve higher grades but also exhibit greater enthusiasm, persistence, and satisfaction with the learning process (Sharma and Sharma, 2018). Teacher practices such as providing constructive feedback, recognizing students’ accomplishments, and setting appropriately challenging yet attainable goals have been found to sustain learners’ intrinsic motivation and reinforce their belief in their academic capabilities (Ahmad et al., 2024; Wang et al., 2019). Beyond cognitive outcomes, these behaviors also nurture affective dimensions of learning, including a sense of belonging and self-worth, which are equally vital for long-term academic engagement.

However, while much of this scholarship addresses general academic motivation, SLA research has demonstrated that motivation in language learning is a distinct construct shaped by unique linguistic, identity-related, and socio-cultural factors (Dörnyei, 2014; Gardner, 2010). Teacher behaviors are particularly influential in this domain because they affect learners’ perceptions of L2 competence, willingness to communicate, linguistic identity, and engagement with the target language community (MacIntyre et al., 1998; Mercer and Dörnyei, 2020). For example, teachers who provide autonomy-supportive feedback and foster positive classroom interactions have been shown to increase learners’ L2 confidence, which directly enhances their readiness to participate in communicative activities, which is a core requirement for successful language development (Saito et al., 2025).

Contrary to the earlier claim that this topic has been understudied, there exists extensive international evidence demonstrating that teacher conduct significantly shapes L2 motivation across diverse cultural contexts (Bai and Wang, 2023; Lamb, 2017; Lan et al., 2023; Papi and Hiver, 2025). Yet, despite this growing body of research, important gaps remain regarding how such teacher behaviors influence learner motivation in foreign language classrooms in underrepresented educational contexts such as Libya. This gap is especially relevant at the secondary level, where students are at a developmental stage characterized by increasing autonomy but also heightened vulnerability to disengagement.

Moreover, in EFL contexts, motivation is often mediated by additional challenges such as limited exposure to authentic language use, curriculum rigidity, and resource constraints. These contextual barriers can amplify the role of teachers as motivational agents who compensate for systemic shortcomings by shaping learners’ affective experiences and linguistic aspirations (Alrabai, 2024; Guilloteaux and Dörnyei, 2008; Henry et al., 2018). In underexplored settings such as Libya, these dynamics are further complicated by socio-cultural expectations, institutional policies, and the lingering effects of systemic challenges within the educational sector. As a result, the relationship between teacher conduct and learner L2 motivation in Libyan secondary schools represents a significant, yet underexamined, area of inquiry. Understanding this relationship is critical not only for improving language learning outcomes but also for informing teacher professional development and policy reforms aimed at fostering more engaging and equitable classrooms.

### 2.2 Cultural and contextual influences in Libya

In Libya, cultural dynamics strongly mediate teacher–student relationships and play a central role in shaping learners’ motivation. The cultural expectation that teachers act as figures of authority and respect can influence both classroom interactions and students’ willingness to engage in learning activities. Teachers who demonstrate cultural sensitivity, acknowledge students’ backgrounds, and respect their values are more likely to foster a sense of belonging and empowerment, thereby enhancing learners’ confidence and engagement (Howard, 2023). In the field of SLA, such culturally responsive practices are particularly important because learners’ attitudes toward the target language are shaped not only by cognitive factors but also by their socio-cultural identities and perceptions of linguistic legitimacy (Norton, 2013; Ushioda, 2020).

By embedding culturally relevant examples and pedagogical practices into their instruction, teachers can create more inclusive and supportive classroom environments where students feel recognized and valued. Research has shown that when L2 instruction reflects learners’ cultural realities, students are more likely to develop positive attitudes toward English and sustain motivation over time (Gay, 2018; Selvi et al., 2022). At the same time, the Libyan education system continues to grapple with systemic challenges that complicate these dynamics. Outdated pedagogical approaches, exam-oriented curricula, and limited access to modern teaching resources are frequently cited obstacles that restrict teachers’ ability to adopt innovative, student-centered practices (Alhodiry, 2016). These constraints often result in classrooms dominated by rote learning, where student motivation is treated as secondary to content delivery.

Such contextual constraints are not unique to Libya; similar patterns have been observed in other EFL contexts where resource scarcity and traditional pedagogical norms impede communicative, autonomy-supportive practices linked to L2 motivation (Lamb, 2017; Tao and Gao, 2021). Consequently, while systemic factors are widely discussed in the literature, the nuanced role of teachers’ day-to-day behaviors—such as the ways they provide feedback, encourage participation, or recognize students’ achievements—has received comparatively little attention. This gap is particularly significant given that Libyan learners often encounter motivational barriers rooted in socio-political instability, restricted exposure to authentic English communication, and conflicting cultural attitudes toward Western languages and values. These sociolinguistic tensions can undermine learners’ L2 confidence and sense of agency, making teachers’ motivational practices even more crucial for sustaining engagement (Mercer, 2021).

In such a context, teachers are not merely content-deliverers but act as cultural mediators who can bridge these gaps and sustain motivation in ways that transcend curriculum constraints. Yet, research exploring how teachers’ behaviors can mitigate or exacerbate these contextual challenges remains scarce. Addressing this oversight is essential not only for advancing theoretical discussions of L2 motivation in peripheral educational settings but also for developing contextually responsive strategies that enhance learner autonomy, participation, and willingness to communicate in English. Such work is vital for improving EFL learning outcomes in Libya and for expanding international motivation research beyond its current concentration in economically privileged or well-resourced settings.

### 2.3 Instructional practices and differentiated approaches

Effective instructional practices are widely recognized as a cornerstone of fostering student motivation in EFL learning. When teachers employ strategies such as differentiated instruction, personalized feedback, and student-centered approaches, they not only enhance learners’ self-efficacy but also strengthen their engagement and persistence in language learning tasks (Pai and Mallya, 2017; Tefera, 2024). Within second language acquisition research, these instructional strategies have been repeatedly identified as catalysts for L2 motivation because they support learners’ psychological needs for autonomy, competence, and relatedness—core components of motivation according to Self-Determination Theory (Deci and Ryan, 1985, 2000; Reeve, 2012). By acknowledging individual accomplishments, providing feedback tailored to students’ strengths and weaknesses, and adapting instruction to different learning preferences, teachers promote a sense of achievement that fuels both immediate participation and long-term intrinsic motivation (Brandmiller et al., 2024). Such practices are especially valuable in EFL contexts, where learners often experience anxiety, linguistic insecurity, and low willingness to communicate; differentiated instructional support can mitigate these challenges by accommodating individual learning trajectories and reducing affective barriers (Oxford, 2016; Loewen and Sato, 2018).

In many international contexts, these practices are associated with improved classroom climates, higher learner autonomy, and deeper investment in language learning. Research demonstrates that when L2 instruction incorporates meaningful choices, collaborative tasks, and scaffolded language use, learners exhibit increased willingness to participate and stronger language-related self-concepts (Dörnyei and Ushioda, 2021; Mercer and Dörnyei, 2020). For instance, student-centered strategies often empower learners to take ownership of their progress, while differentiated instruction ensures that diverse abilities and backgrounds are addressed equitably. Together, such practices help balance cognitive demands with affective support, leading to more sustainable learning outcomes.

Despite this robust global evidence, the extent to which these strategies are integrated into Libyan secondary EFL classrooms remains unclear. Existing studies tend to emphasize systemic challenges such as resource limitations and curriculum rigidity (Pathan and Marayi, 2016), but seldom explore whether teachers adapt instructional methods to meet learners’ individual needs. Limited empirical research has examined how Libyan teachers respond to learner diversity, structure communicative tasks, or personalize feedback—practices that are central to sustaining L2 motivation. This lack of localized evidence raises questions about the transferability of global best practices to the Libyan context. Without empirical insights into how Libyan teachers implement, modify, or even resist differentiated and student-centered practices, it is difficult to assess their potential impact on learner motivation and engagement. Addressing this gap is crucial because instructional practices that are effective in Western, well-resourced environments cannot be assumed to operate identically in post-conflict education systems characterized by limited professional development opportunities, traditional classroom norms, and societal ambivalence toward English (Tao and Gao, 2021). Identifying contextually appropriate instructional strategies that bridge global pedagogical principles with local realities represents a necessary step toward improving L2 learning outcomes in Libya.

### 2.4 Teacher perceptions and student outcomes

Teachers’ perceptions of their students’ motivation and engagement also shape classroom interactions. Educators often adjust their instructional strategies based on how motivated they believe their students to be (Eskelä-Haapanen et al., 2021; Havik and Westergård, 2020). When teachers perceive learners as engaged, they tend to provide greater autonomy, more structured learning opportunities, and higher expectations (Skinner and Belmont, 1993; Wang and Hofkens, 2020). Conversely, when disengagement is attributed to low ability rather than situational factors, teachers may resort to negative feedback and reduced support (Guskey and Link, 2019). Recent studies show that students are highly aware of these differentiated treatments, and they respond accordingly. For example, Yang (2021) found that when teachers conveyed high expectations through encouragement and constructive feedback, students internalized these beliefs and increased their involvement. Conversely, when teachers demonstrated favoritism or compared students negatively, learners became disengaged. Such findings underscore the importance of teachers’ attitudes in shaping not only achievement but also the socio-psychological climate of the classroom.

Within the domain of second language learning, teacher perceptions hold added significance because students’ L2 motivation and willingness to communicate are directly influenced by the messages they receive about their linguistic potential (Mercer, 2021; Papi and Hiver, 2025). Teachers who perceive learners as capable language users are more likely to create opportunities for meaningful interaction, scaffold communicative tasks, and provide autonomy-supportive feedback—practices that enhance L2 confidence and identity investment (Dörnyei, 2014; Henry and Thorsen, 2018). In contrast, perceptions of linguistic inadequacy can result in restrictive teaching practices that reduce L2 participation and reinforce learners’ anxiety or self-doubt (Xi, 2019). Thus, teacher perceptions do not merely reflect learners’ motivation; they construct it by shaping the opportunities, expectations, and affective conditions under which language learning occurs.

Despite these insights, few studies have systematically examined how teacher perceptions and behaviors interact to influence motivation in Libyan EFL contexts. Existing Libyan research (e.g., Pathan and Marayi, 2016) often lacks methodological rigor, relying on small-scale qualitative approaches without validated instruments. Moreover, no studies to date have examined teacher perceptions in relation to empirically measured instructional practices, leaving a critical gap in understanding how beliefs translate into pedagogical choices that shape L2 motivation. This methodological weakness further emphasizes the need for more robust, quantitative investigations.

In this study, it is important to distinguish between two related but separate constructs. First, teacher motivational behaviors (instructional practices) refer to the strategies and classroom actions teachers report using (e.g., feedback, interaction, autonomy support). Second, teacher-perceived learner motivational outcomes refer to teachers’ professional judgments about how learners respond motivationally (e.g., perceived learner motivation, engagement, and confidence). Thus, the present research does not treat learner motivation as a directly measured internal learner state; rather, it examines how teachers’ self-reported practices relate to their perceptions of learners’ motivational experiences.

### 2.5 Theoretical framework

This study is anchored in two key motivational theories: Self-Determination Theory (SDT) and Teacher Expectancy Theory (TET), both of which provide valuable perspectives for analyzing how teacher behaviors influence student motivation in EFL contexts.

SDT, initially developed by Deci and Ryan (1985) and later expanded to educational settings, argues that learners’ intrinsic motivation is fostered when three fundamental psychological needs are satisfied: autonomy, competence, and relatedness. In the classroom, teachers can promote autonomy by offering choices and encouraging learner agency, support competence through constructive feedback and scaffolded learning, and enhance relatedness by building warm, supportive relationships with students. In EFL contexts, where learners often experience anxiety and limited opportunities for authentic language use, satisfying these needs is closely linked to sustained L2 motivation and willingness to communicate. In Libya, where traditional teacher-centered instruction remains dominant (Pathan and Marayi, 2016), the degree to which teachers’ practices fulfill these needs becomes particularly important. SDT therefore provides a useful lens for examining whether classroom behaviors contribute to internalized, self-sustaining motivation among secondary learners.

TET, introduced by Rosenthal and Jacobson (1968), highlights the powerful effect of teachers’ expectations on student outcomes. When educators hold positive expectations and communicate them through encouragement, praise, and high but achievable standards, students are more likely to believe in their own capabilities and strive to meet those expectations. Conversely, negative expectations can undermine confidence, leading to disengagement and lower achievement. In EFL settings where linguistic confidence is fragile, teacher expectations may directly influence learners’ beliefs about their language abilities and their willingness to participate—a dynamic heightened in Libya, where resource limitations and systemic challenges often restrict learners’ opportunities.

Taken together, these two theories provide a complementary framework for understanding the dynamics between teacher behaviors, instructional practices, and learner motivation. SDT highlights the internal psychological mechanisms that sustain motivation, while TET emphasizes the interpersonal and relational dimension of teacher–student interactions. Using these frameworks, this study seeks to explore how Libyan secondary school EFL teachers’ practices contribute to or hinder learner motivation, confidence, and engagement.

## 3 Research design

This study employed a quantitative, cross-sectional survey design to investigate the relationship between secondary school teachers’ instructional practices and their perceptions of learners’ L2 motivation, confidence, and engagement in Libyan EFL classrooms. A survey design was considered appropriate because the aim was not to test the causal efficacy of particular instructional styles, but rather to capture teachers’ self-reported practices and their perceived influence on learners’ motivational outcomes, an approach widely used in L2 motivation research (Dörnyei and Taguchi, 2009).

The quantitative design allowed for the systematic collection of standardized data from a relatively large sample of secondary school teachers, enabling the use of descriptive and inferential statistical analyses such as t-tests, ANOVA, correlations, and regression models. These analyses were used to examine differences across demographic groups and to identify the predictive strength of instructional practices on teacher-reported learner motivation, thereby moving beyond purely descriptive accounts and providing empirical evidence of associations among variables.

Furthermore, adopting a cross-sectional approach ensured that data were collected at a single point in time from multiple schools, making it possible to compare diverse contexts while avoiding the resource demands of a longitudinal design. Survey-based methodologies are well established in studies examining teacher behaviors and L2 motivation, particularly when investigating large populations across educational settings. By grounding the research in a quantitative, cross-sectional framework, the study contributes empirical evidence from an underexplored context—Libyan secondary schools—while ensuring methodological rigor aligned with the research questions.

### 3.1 Participants and sampling

The target population of this study consisted of secondary school EFL teachers in Libya. A total of 250 teachers participated, representing a range of demographic and professional backgrounds. English is taught as a compulsory subject in Libyan secondary schools, and although precise nationwide statistics are not publicly available, English teachers constitute a substantial proportion of the national teaching workforce at this level.

A multistage sampling strategy was employed to ensure both feasibility and diversity. First, five secondary schools were purposively selected based on variation in school size, regional location, and available resources, ensuring that different educational environments were represented. This selection reflects typical characteristics of Libyan public secondary education, where English instruction is mandated but unevenly supported in terms of resources and teacher preparation. Within these schools, teachers were randomly invited to participate, reducing the potential for selection bias and enhancing representativeness. This combination of purposive school selection and random teacher recruitment aligns with best practices for educational survey research seeking contextual relevance without sacrificing analytic rigor. Table 1 summarizes the demographic characteristics of the participants.

**Table 1:** Demographic characteristics of the participants.

Characteristic	Category	Percentage
Gender	Male	60%
	Female	40%
Age group	20–30 years	25%
	31–40 years	40%
	41–50 years	25%
	Above 50 years	10%
Teaching experience	Less than 5 years	20%
	5–10 years	35%
	More than 10 years	45%
Educational qualification	Bachelor’s degree	50%
	Master’s degree	40%
	Doctorate (PhD)	10%

This sample reflects a diverse cross-section of Libyan secondary school EFL teachers in terms of gender, age, professional experience, and academic qualifications. However, while the sample of 250 teachers is substantial, it does not encompass the entire population of Libyan EFL teachers; therefore, the findings should be interpreted cautiously and not assumed to generalize nationally. Such diversity nonetheless strengthens the external validity of the study and supports the applicability of the results across a variety of secondary school settings in Libya.

### 3.2 Instrument

Data were collected using a structured 30-item questionnaire designed to measure teachers’ instructional practices and their perceptions of learners’ L2 motivation, confidence, and engagement in Libyan secondary schools. The instrument employed a five-point Likert scale ranging from 1 = strongly disagree to 5 = strongly agree. Items were grouped into ten thematic categories, each reflecting a distinct instructional dimension (e.g., teacher feedback, classroom interaction, student autonomy), with three items per category. This structure allowed for the systematic exploration of a wide range of teaching practices while maintaining coherence with the study’s objectives. The development of the questionnaire was guided by the study’s theoretical framework, particularly Self-Determination Theory (Deci and Ryan, 1985, 2000) and Teacher Expectancy Theory (Rosenthal and Jacobson, 1968). These perspectives informed the choice of constructs, ensuring that the items addressed core elements such as autonomy support, competence feedback, relatedness, and the influence of teacher expectations on learner motivation.

To establish content validity, the instrument was reviewed by a panel of three educational experts from a Libyan university, who assessed the relevance, clarity, and alignment of the items with the study’s research questions and theoretical underpinnings. Following expert feedback, the questionnaire was refined for clarity and appropriateness to the local context. A pilot study was then conducted with 20 secondary school teachers, which provided preliminary evidence of item clarity and usability.

Construct validity was examined using Exploratory Factor Analysis (EFA) in SPSS. Preliminary tests confirmed the suitability of the data for factor analysis: the Kaiser–Meyer–Olkin (KMO) measure exceeded the recommended threshold of 0.60, and Bartlett’s Test of Sphericity was statistically significant (*p* < 0.001), indicating adequate inter-item correlations. The EFA results suggested that the extracted factors explained approximately 32.9% of the total variance, supporting the multidimensional structure of the instrument. Although the explained variance was modest, it was acceptable for an exploratory study of this scope, particularly given the diverse nature of the instructional categories examined.

The reliability of the instrument was assessed using Cronbach’s alpha coefficients. Internal consistency values exceeded the commonly accepted threshold of 0.70 for most subscales, indicating satisfactory reliability. These results suggest that teachers’ reports on learners’ motivational outcomes were captured consistently through perceptions rather than direct learner measures, which aligns with survey-based motivation research in EFL contexts (Dörnyei and Taguchi, 2009).

It is important to note that the questionnaire does not measure learners’ motivation, engagement, or confidence directly. Instead, these constructs are operationalised through teachers’ perceptions, captured by items asking respondents to evaluate how their instructional practices influence learners’ motivational experiences. This teacher-report approach is consistent with perception-based research designs in educational psychology and L2 motivation research.

### 3.3 Data collection procedure

The questionnaire was administered via Google Forms, chosen for its accessibility and ease of use within the Libyan context. The platform facilitated broad distribution through widely used communication channels such as email and WhatsApp, ensuring convenience for participants and efficient management of the data collection process. To enhance clarity and minimize response errors, the survey was accompanied by written instructions that outlined the study’s purpose, explained the structure of the questionnaire, and provided guidance on how to interpret the Likert-scale items. Participation was voluntary, and no personally identifiable information was collected. Teachers could complete the survey at their convenience within the two-week data collection period.

A total of 250 valid responses were obtained. The response rate was considered adequate for quantitative analysis, given the scope of the study and the multistage sampling procedure. Online administration also minimized logistical costs and allowed direct export of responses to SPSS for analysis.

### 3.4 Ethical considerations

This study was conducted in accordance with recognized ethical standards for research involving human participants. Ethical approval was obtained from the Research Ethics Committee of the researchers’ institution (Decision no: EKK23-24/015/02), ensuring that all procedures adhered to institutional and international guidelines for educational research. Participation was voluntary, and informed consent was obtained from all participants prior to data collection. The consent form provided information about the purpose of the study, the nature of participation, confidentiality measures, and the participants’ right to withdraw at any time without penalty. To protect privacy, no identifying information was collected, and responses were anonymized. The study posed minimal risk to participants, as the survey focused exclusively on professional teaching practices and did not involve sensitive personal or health-related information. The potential benefits outweighed the risks, particularly in contributing evidence to an underrepresented educational context.

### 3.5 Data analysis

Data were exported from Google Forms into SPSS (version 25) for analysis. Prior to conducting statistical tests, the dataset was screened for missing values. In cases where more than 20% of a respondent’s items were unanswered, their data were excluded using listwise deletion. For minor missing responses, mean substitution was applied to maintain dataset integrity (Field, 2024). After data cleaning, 250 valid responses were retained for analysis.

The analysis proceeded in several stages. First, descriptive statistics (means and standard deviations) were computed for the ten instructional categories. Internal consistency reliability was assessed using Cronbach’s alpha, with values ≥ 0.70 considered acceptable (Tavakol and Dennick, 2011). Next, inferential statistical tests were performed to examine group differences and relationships between variables in line with the research questions. Specifically:

- Independent samples t-tests examined differences in reported instructional practices based on gender and years of teaching experience.
- One-way ANOVAs compared responses across age groups and academic qualifications, with Tukey’s HSD used where appropriate.
- Pearson correlation coefficients evaluated relationships between instructional practices (e.g., teacher feedback, classroom interaction, autonomy support) and teacher-reported perceptions of learners’ motivation, engagement, and confidence.
- Multiple regression analysis assessed the predictive power of instructional practices on teacher-reported learner motivation, with teacher feedback, classroom interaction, and autonomy support entered as predictors in line with prior research.

It is important to note that learner motivation, engagement, and confidence were not measured directly from students, but were examined exclusively through teachers’ perceptions in a teacher-report correlational design. Instead, these constructs were operationalised through teachers’ perceptions, captured via questionnaire items that asked respondents to evaluate the extent to which their instructional practices influenced learners’ motivational experiences. Thus, all motivational outcomes in this study reflect teacher-reported evaluations, rather than learner-generated data. Motivational outcomes were calculated by averaging teachers’ responses to the Likert-scale items corresponding to each construct (motivation, engagement, and confidence), thereby producing composite scores that could be used in the correlational analyses. Higher mean scores reflected stronger teacher-perceived learner motivation, engagement, or confidence.

The combination of descriptive and inferential analyses ensured a coherent profile of the sample and a rigorous examination of the relationships between instructional practices and motivational outcomes. Results were interpreted at a significance threshold of *p* < 0.05.

## 4 Findings

This section presents the results of the statistical analyses conducted to examine Libyan secondary school teachers’ perceptions of instructional practices and their reported influence on learners’ L2 motivation, confidence, and engagement. The analysis is organized into four parts: (a) descriptive statistics and reliability estimates for the instructional categories, (b) group differences based on demographic characteristics, (c) correlations among instructional practices and motivational outcomes, and (d) regression analyses identifying the strongest predictors of learner motivation. As the study focuses on teachers’ perceptions rather than direct learner measures, the results reflect teachers’ interpretations of how their instructional practices shape learners’ motivational experiences, a common approach in survey-based L2 motivation research. Together, these findings provide both an overview of prevailing teaching practices and deeper insights into the relationships between teacher behaviors and learners’ motivational experiences.

### 4.1 Descriptive statistics and reliability

Descriptive statistics and reliability estimates were computed for the ten instructional categories measured in the study. Table 2 presents the means, standard deviations, and Cronbach’s alpha coefficients for each subscale.

**Table 2:** Descriptive statistics and reliability of instructional categories (*N* = 250).

Instructional category	*M*	SD	Cronbach’s α
Teacher feedback	4.21	0.62	0.74
Classroom interaction	4.15	0.59	0.72
Autonomy support	4.08	0.65	0.71
Goal-setting strategies	3.98	0.67	0.70
Use of engaging materials	3.92	0.68	0.68
Differentiated instruction	3.85	0.71	0.66
Recognition of achievement	3.80	0.73	0.64
Encouraging peer collaboration	3.78	0.70	0.65
Classroom management	3.72	0.75	0.62
Cultural responsiveness	3.65	0.79	0.63

M, Mean; SD, Standard deviation.

The results indicate that teachers reported the highest levels of practice in the areas of teacher feedback (*M* = 4.21), classroom interaction (*M* = 4.15), and autonomy support (*M* = 4.08). These practices correspond to motivationally salient behaviors identified in L2 research, as they promote competence, relatedness, and learner agency within the classroom. In contrast, cultural responsiveness (*M* = 3.65) and classroom management (*M* = 3.72) received the lowest mean scores, suggesting that these dimensions are less consistently embedded in teachers’ instructional routines and may represent priority areas for targeted professional development.

The reliability analysis showed that most subscales reached acceptable levels of internal consistency (*α* ≥ 0.70). However, some categories—including classroom management (*α* = 0.62), cultural responsiveness (*α* = 0.63), and recognition of achievement (*α* = 0.64)—fell below the recommended threshold. This indicates that items within these subscales were not as closely related as expected, possibly because teachers interpret or enact these practices in varied ways across different school contexts. While the overall instrument demonstrated satisfactory reliability, these dimensions may require refinement in future studies to improve construct clarity and ensure more stable measurement across respondents.

### 4.2 Group differences

To examine whether perceptions of instructional practices varied across demographic groups, a series of independent-samples t-tests and one-way ANOVAs were conducted. Table 3 summarizes the key results.

**Table 3:** Group differences in instructional practices (*t*-tests and ANOVAs).

Variable	Comparison groups	Test statistic	*p*-value	Significant findings
Gender	Male (*n* = 150) vs. Female (*n* = 100)	*t*(248) = 1.12	0.26	Not significant
Teaching experience	< 5 yrs., 5–10 yrs., > 10 yrs	*F*(2, 247) = 3.42	0.034	< 5 yrs. > > 10 yrs
Age group	20–30, 31–40, 41–50, 50+	*F*(3, 246) = 4.21	0.007	Younger > Older
Educational qualification	Bachelor’s, Master’s, PhD	*F*(2, 247) = 1.56	0.21	Not significant

The results showed no significant gender differences in teachers’ reported practices, suggesting that male and female teachers perceived their use of motivational strategies similarly. However, significant effects were observed for teaching experience and age. Teachers with less than 5 years of experience reported significantly higher use of motivational practices compared to those with over 10 years of experience. Similarly, younger teachers (20–30 years old) reported higher levels of classroom interaction and use of engaging materials compared to older age groups. No significant differences were found across educational qualification levels.

These results suggest that younger and less experienced teachers may be more inclined to adopt innovative or student-centered practices, whereas more experienced teachers may rely more heavily on traditional instructional approaches. One possible explanation is that younger and less experienced teachers are more recently trained and therefore more familiar with contemporary student-centred and technology-assisted pedagogies, which emphasise interaction and motivational support. In contrast, more experienced teachers may rely more heavily on traditional, teacher-fronted approaches shaped by earlier training and long-standing institutional practices, which may reduce the frequency of such strategies.

### 4.3 Correlations

Pearson product–moment correlation coefficients were calculated to examine the strength and direction of the relationships between instructional practices and teacher-reported learner motivation, engagement, and confidence. Table 4 presents the results.

**Table 4:** Correlations between instructional practices and motivational outcomes (*N* = 250).

Variable	Learner motivation	Learner engagement	Learner confidence
Teacher feedback	0.62**	0.55**	0.49**
Classroom interaction	0.58**	0.61**	0.52**
Autonomy support	0.54**	0.49**	0.57**
Goal-setting strategies	0.41**	0.38**	0.35**
Engaging materials	0.46**	0.43**	0.37**
Differentiated instruction	0.39**	0.36**	0.34**
Recognition of achievement	0.42**	0.40**	0.33**
Peer collaboration	0.44**	0.47**	0.36**
Classroom management	0.28*	0.30*	0.25*
Cultural responsiveness	0.31*	0.27*	0.29*

*p < 0.05, **p < 0.01.

The results indicated that the strongest positive correlations with learner motivation were observed for teacher feedback (*r* = 0.62, *p* < 0.01), classroom interaction (*r* = 0.58, *p* < 0.01), and autonomy support (*r* = 0.54, *p* < 0.01). Similarly, classroom interaction was most strongly correlated with learner engagement (*r* = 0.61, *p* < 0.01), while autonomy support showed the strongest correlation with learner confidence (*r* = 0.57, *p* < 0.01). By contrast, classroom management and cultural responsiveness were only weakly correlated with motivational outcomes, though still statistically significant. This suggests that while these practices are present, they may not exert a direct or salient motivational influence in their current form.

These findings highlight the pivotal role of feedback, interaction, and autonomy support in shaping learners’ motivational experiences, reinforcing the idea that practices which satisfy learners’ needs for competence, relatedness, and autonomy exert the strongest motivational pull in EFL settings, consistent with the assumptions of Self-Determination Theory (Ryan and Deci, 2020).

### 4.4 Regression analysis

To identify the instructional practices that most strongly predicted learner motivation, a multiple regression analysis was conducted with teacher feedback, classroom interaction, and autonomy support entered as predictors. These variables were selected based on their high correlations with learner motivation in the previous analysis. Table 5 presents the results.

**Table 5:** Multiple regression analysis predicting learner motivation (*N* = 250).

Predictor	*β*	*t*	*p*
Teacher feedback	0.45	7.82	< 0.001
Classroom interaction	0.28	5.14	< 0.001
Autonomy support	0.22	4.36	< 0.001

Model statistics: *R*^2^ = 0.42, Adjusted *R*^2^ = 0.41, *F*(3, 246) = 58.96, *p* < 0.001.

The model explained approximately 42% of the variance in learner motivation, indicating a moderate-to-strong predictive relationship. Among the predictors, teacher feedback emerged as the strongest predictor (*β* = 0.45, *p* < 0.001), followed by classroom interaction (*β* = 0.28, *p* < 0.001) and autonomy support (*β* = 0.22, *p* < 0.001).

These results underscore the central role of feedback in shaping learners’ motivational experiences, while also highlighting the importance of interactive teaching and opportunities for learner autonomy. Together, these findings align with the principles of Self-Determination Theory (Ryan and Deci, 2020), which emphasizes the fulfillment of students’ psychological needs for competence, relatedness, and autonomy as key drivers of motivation.

### 4.5 Summary of findings

In summary, the findings indicate that Libyan secondary school teachers reported frequent use of practices such as feedback, classroom interaction, and autonomy support, which were also found to be the most strongly associated with learners’ motivation, confidence, and engagement. While group differences were limited, younger and less experienced teachers reported higher reliance on motivational strategies compared to their older and more experienced counterparts. Correlational analyses further highlighted the central role of interactive and student-centered practices, while regression analysis confirmed that teacher feedback was the strongest predictor of learner motivation. Although some instructional categories demonstrated lower internal consistency, the overall results underscore the importance of fostering supportive, communicative, and autonomy-enhancing classroom environments to sustain learner motivation in the Libyan secondary education context.

## 5 Discussion

This study examined the instructional practices employed by Libyan secondary school EFL teachers and their perceptions of how these practices influence learners’ motivation, engagement, and confidence. Drawing on a sample of 250 teachers across five schools, the analysis sought to identify which teaching strategies were most strongly associated with positive motivational outcomes and to explore whether teachers’ demographic characteristics shaped their reported practices.

The findings demonstrated that teacher feedback, classroom interaction, and autonomy support were the most frequently reported practices and the strongest predictors of L2 motivation. Among these, feedback emerged as the single most powerful contributor, underscoring its central role in fostering students’ confidence and perceived competence. Younger and less experienced teachers were more likely to adopt student-centered and interaction-oriented approaches, while more experienced teachers reported comparatively lower use of motivational strategies. By contrast, practices such as cultural responsiveness and classroom management received lower ratings and demonstrated weaker reliability, suggesting areas where further refinement of both practice and measurement is needed. Together, these results highlight the importance of supportive, communicative, and autonomy-enhancing environments for sustaining learners’ engagement in EFL classrooms in Libya.

### 5.1 Linking findings to literature and theory

Because this study was situated in an EFL context, the motivational constructs and instructional practices examined here are directly tied to learners’ experiences in second language classrooms.

The central role of teacher feedback in predicting learner motivation is consistent with SDT, which identifies competence as a basic psychological need essential for sustaining intrinsic motivation (Deci and Ryan, 2000). Feedback provides students with recognition of progress and guidance for improvement, thereby strengthening their sense of competence. This aligns with the socio-educational assumption that L2 confidence is shaped by perceived progress and teacher validation, echoing TET’s emphasis on how teachers’ expectations influence learners’ self-beliefs (Rosenthal and Jacobson, 1968).

Similarly, the strong associations between classroom interaction and motivational outcomes resonate with research highlighting the role of relatedness in language learning. Studies have shown that learners are more engaged when teachers create interactive environments that encourage dialogue, collaboration, and mutual respect (Reeve, 2006; Wang and Hofkens, 2020). In L2 contexts, interaction is particularly salient, as opportunities to use the target language directly strengthen learners’ willingness to communicate and sustain their motivational trajectories.

The importance of autonomy support further reinforces SDT’s claim that a sense of choice and independence is critical for internalized motivation. Teachers who involve learners in decision-making or encourage independent problem-solving contribute to ownership of learning. This finding is consistent with SLA research positioning learner agency as a key determinant of L2 investment and persistence.

By contrast, the relatively lower scores and weaker reliability of cultural responsiveness and classroom management reflect persistent gaps in Libyan EFL practice. While cultural integration has been linked to stronger learner identity and belonging (Gay, 2018), its role may be limited locally due to insufficient training. Classroom management, although necessary for order, does not directly support psychological needs central to L2 motivation, which may explain its weaker associations empirically.

### 5.2 Contributions of the study

This study makes several contributions to the understanding of motivational practices in EFL contexts, particularly in underrepresented educational settings such as Libya. First, in relation to RQ1, the findings highlight the significance of teacher feedback, classroom interaction, and autonomy support in shaping learners’ motivational experiences, extending SDT and TET into a context not typically featured in global SLA research. In relation to RQ2, the study identifies instructional practices that teachers perceive as most effective for enhancing learners’ motivation and confidence.

More broadly, the study contributes to comparative educational research by focusing on a North African context seldom represented in motivational literature, which has historically centered on East Asian and European settings. By identifying both strengths—such as interaction and feedback—and weaknesses—such as cultural responsiveness—the findings offer evidence that can inform teacher development initiatives and curriculum design in Libya and similar EFL environments.

### 5.3 Limitations of the study

Although this study provides valuable insights, several limitations should be acknowledged. First, the study relied exclusively on self-reported data, which may be subject to social desirability or subjective interpretation. Second, although the questionnaire demonstrated acceptable reliability overall, several subscales (e.g., cultural responsiveness, classroom management) fell below the recommended threshold, indicating that further refinement is needed. Third, the sample, although diverse, was limited to five schools and may not represent all Libyan secondary teachers. Finally, the cross-sectional design prevents causal interpretation of relationships. Longitudinal or intervention-based studies would be required to verify whether specific instructional practices lead to sustained changes in L2 motivation.

### 5.4 Implications and future research

The findings carry several implications. First, professional development programs should prioritize feedback, interaction, and autonomy support, as these practices were identified as the most influential motivational drivers. Second, the lower ratings for cultural responsiveness point to the value of integrating culturally relevant pedagogy into teacher training. Third, policymakers should encourage student-centered and autonomy-supportive approaches rather than traditional teacher-dominated models. Future research should employ mixed-methods and longitudinal designs and include confirmatory factor analysis to strengthen the psychometric properties of the instrument. Expanding samples beyond the current schools would also enhance external validity and allow clearer generalization across Libya.

## 6 Conclusion

This study examined the instructional practices of Libyan secondary school EFL teachers and their influence on learners’ L2 motivation, engagement, and confidence. The findings underscored the importance of teacher feedback, classroom interaction, and autonomy support as the most frequently used and effective motivational practices. In contrast, cultural responsiveness and classroom management were rated lower, suggesting areas for improvement in teacher preparation and curriculum design.

By situating the results within SDT and TET, the study demonstrates how teachers’ behaviors can directly shape learners’ psychological needs and expectations, thereby influencing their willingness to invest in language learning. Although limited by self-report measures and sampling constraints, the study contributes important empirical evidence from a context rarely examined in SLA research.

Ultimately, the study provides practical directions for improving EFL instruction in Libya and contributes to broader discussions on how motivational theories can be applied in diverse educational contexts. Future research integrating qualitative and longitudinal approaches will be essential for advancing our understanding of how motivational practices evolve and sustain L2 engagement over time.

## Data Availability

The raw data supporting the conclusions of this article will be made available by the authors, without undue reservation.
