# Supplementary material for: Teacher motivational practices and their perceived influence on L2 motivation in Libyan secondary EFL classrooms
Source: Front Psychol. 2026 Feb 18;17:1701372. doi: 10.3389/fpsyg.2026.1701372 (PMC12956704; doi:10.3389/fpsyg.2026.1701372)
Supplement: Supplementary file 1 [file Supplementary_file_1.docx]

**APPENDIX**

**Teacher Questionnaire**

*Instructional Practices and Learner Motivation in Secondary EFL Classrooms*

**Instructions:**
Please indicate the extent to which you agree with the following statements about your teaching practices and classroom experiences. Responses are on a 5-point Likert scale:
1 = Strongly Disagree, 2 = Disagree, 3 = Neutral, 4 = Agree, 5 = Strongly Agree.

| **1. Feedback** | **Strongly Disagree** | **Disagree** | **Neutral** | **Agree** | **Strongly Agree** |
| --- | --- | --- | --- | --- | --- |
| 1. I provide constructive comments on my students’ assignments. |  |  |  |  |  |
| 2. I give timely feedback that helps students improve their performance. |  |  |  |  |  |
| 3. I acknowledge both my students’ strengths and areas for improvement. |  |  |  |  |  |
| **2. Classroom Interaction** |  |  |  |  |  |
| 4. I encourage active participation during lessons. |  |  |  |  |  |
| 5. I create an environment where students feel comfortable asking questions. |  |  |  |  |  |
| 6. I use discussions to make lessons more engaging. |  |  |  |  |  |
| **3. Autonomy Support** |  |  |  |  |  |
| 7. I allow students to make choices in learning activities. |  |  |  |  |  |
| 8. I encourage students to take responsibility for their own learning. |  |  |  |  |  |
| 9. I support independent thinking and decision-making. |  |  |  |  |  |
| **4. Classroom Management** |  |  |  |  |  |
| 10. I maintain discipline in a fair and respectful manner. |  |  |  |  |  |
| 11. I keep the classroom environment well-organized and conducive to learning. |  |  |  |  |  |
| 12. I manage disruptions effectively so learning is not interrupted. |  |  |  |  |  |
| **5. Cultural Responsiveness** |  |  |  |  |  |
| 13. I show respect for my students’ cultural backgrounds. |  |  |  |  |  |
| 14. I include examples in lessons that reflect students’ cultural experiences. |  |  |  |  |  |
| 15. I ensure students’ cultural identities are acknowledged and valued. |  |  |  |  |  |
| **6. Instructional Clarity** |  |  |  |  |  |
| 16. I explain concepts clearly and thoroughly. |  |  |  |  |  |
| 17. I communicate lesson objectives at the start of class. |  |  |  |  |  |
| 18. I ensure students understand what is expected in assignments and activities. |  |  |  |  |  |
| **7. Motivation Strategies** |  |  |  |  |  |
| 19. I use activities that make learning English interesting. |  |  |  |  |  |
| 20. I motivate students by using a variety of teaching methods. |  |  |  |  |  |
| 21. I aim to inspire students to stay engaged with lesson content. |  |  |  |  |  |
| **8. Recognition of Achievement** |  |  |  |  |  |
| 22. I praise students for their progress and accomplishments. |  |  |  |  |  |
| 23. I recognize students’ efforts when they work hard in class. |  |  |  |  |  |
| 24. I celebrate students’ achievements in ways that encourage continued effort. |  |  |  |  |  |
| **9. Instructional Variety** |  |  |  |  |  |
| 25. I use different teaching methods (e.g., group work, projects, media). |  |  |  |  |  |
| 26. I vary classroom activities to avoid repetition. |  |  |  |  |  |
| 27. I use different types of materials to support learning. |  |  |  |  |  |
| **10. Teacher–Student Relationship** |  |  |  |  |  |
| 28. I treat students with respect and fairness. |  |  |  |  |  |
| 29. I provide support when students face difficulties. |  |  |  |  |  |
| 30. I show interest in my students’ progress and success. |  |  |  |  |  |
